# New partnerships among single older adults: a Q methodology study

**DOI:** 10.1186/s12877-019-1091-5

**Published:** 2019-03-06

**Authors:** Su-Fei Huang, Chiu-Mieh Huang, Shueh-Fen Chen, Li-Ting Lu, Jong-Long Guo

**Affiliations:** 1 Department of Senior Citizen Service, Mackay Junior College of Medicine, Nursing, and Management, Taipei, Taiwan; 2 Institute of Clinical Nursing, School of Nursing, National Yang-Ming University, Taipei, Taiwan; 3 Department of Senior Citizen Care and Welfare, Ching Kuo Institute of Management & Health, Keelung, Taiwan; 4 Department of Nursing, University of Kang Ning, Taipei, Taiwan; 5 Department of Health Promotion and Health Education, National Taiwan Normal University, Taipei, Taiwan

**Keywords:** Older adults, Partnerships, Q methodology, Single status

## Abstract

**Background:**

The social structure is changing with an increase in the ratio of the older population, resulting in a growing number of older people being faced with singlehood. This study identified and described single older adults’ differing perspectives on new relationships.

**Method:**

We used a Q methodology approach for data collection and analysis, following in-depth interviews with 10 participants. Q statements were developed through content analysis of the interview data, which were then subjected to Q sorts performed by 49 older adults. A factor analysis was then completed on the collected data using PQ Method software.

**Results:**

Five factors regarding common attitudes toward pursuing a new partner, which accounted for 53% of the total variance, were obtained in the final model: (1) being single, a companion, and already acquainted with the other person/potential partner; (2) high spiritual compatibility and a caring disposition; (3) an emphasis on physical intimacy and companionship; (4) easily influenced by others’ comments and highly concerned about being alone; and (5) physical and financial independence.

**Conclusions:**

Clustering older adults according to their attitudes can help in acknowledging their expectations about new relationships in later life.

**Implications:**

Practitioners can engage in successful consultations based on the recognition.

**Electronic supplementary material:**

The online version of this article (10.1186/s12877-019-1091-5) contains supplementary material, which is available to authorized users.

## Background

As a population ages, the socio-demographic structure is characterized by the increase in the number of older adults. The impact of this change will be particularly pronounced when the majority of the “baby boomer” generational cohort (i.e., adults born between 1946 and 1964) enters old age. This global aging trend is a byproduct of extended life expectancies resulting from medical advances and is a social and economic issue worldwide. In 2015, the average life expectancy of the global population at birth was 71.4 years, and the proportion of adults aged over 60 years was expected to have a twofold increase by 2050 [1, 2]. This is expected to lead to a significant social change.

Several previous studies have indicated that older adults experience varying levels of loneliness, but the general trend is an increasing rate of these types of feelings [3–5]. De Jong Gierveld and Fokkema [6] examined the concept of loneliness prevention, wherein its key point is to create and maintain the quality and quantity of a personal relationships network. Establishing a new romantic relationship is also considered an active method for preventing loneliness in later life [7].

Dating has become a common activity in later life as the proportion of older adults who are single continues to rise. The analysis of Brown and Shinohara [8], based on the United States’ 2005–2006 National Social Life, Health, and Aging Project data, found that roughly 14% of single older adults were in a dating relationship. Dating in later life has been linked positively to health and general well-being; for example, a previous study revealed that daters had a higher social advantage, better health, and reported more social connectedness, compared to non-daters [9]. Older adults have also shown a desire for companionship to avoid the status of singlehood [10, 11]. Elderly who desire a new romantic partnership and actively dated reported fewer symptoms of depression [9, 12] as their fundamental relational needs were met, thereby reducing loneliness and providing meaning in later life [13].

Older adults’ attitudes toward pursuing a new partner vary according to their gender and age; for example, more male older adults disclose a desire for a new partner than females [8], and because the desire to date decreases with age, it is more often a characteristic of older adults under the age of 70 [8, 13]. Moreover, these partnerships involved multiple and complex meanings, as having a partner might not guarantee happiness, but partnership can assist in constructing a self-image of feeling young [14, 15]. In addition, finding a partner through dating can provide emotional and sexual intimacy [7, 12, 16, 17] and an opportunity to establish a mutually beneficial relationship that can fulfill interpersonal relationship needs and reduce feelings of loneliness [13, 15, 18, 19]. Finding and having a new partner is a complex process that is influenced by one’s personal point of view toward dating, parent–child relationships, social norms, and culture [17, 20]. The act of dating in an effort to engage in partnership is different from lifelong marriage [12, 15].

Examining older adults’ perspectives on partnerships would assist in clinicians’ better understanding of older adults’ needs surrounding a variety of critical issues in later life. The Q methodology can identify the heterogeneity of subjectivity within recruited samples through the process of “Q sorts.” This approach integrates qualitative and quantitative research methods by strengthening conceptual categorization with the quantification of patterned subjectivities [21, 22]. By depending substantially on subjectivity, older adults would be able to disclose their perspectives about partnerships more freely, without the constraints imposed by quantitative survey methods. The Q methodology has been broadly applied in health-related research, such as ascertaining employees’ attitudes toward the resource requirements for breastfeeding [23], effective retention strategies for mid-career critical-care nurses [24], and older adults’ perspectives and beliefs on preventing falls [25]. It has also been applied to investigate the subjective definition of love among 59 British women aged 18–61 years [26]. Thus, the Q methodology has been applied in this present study in an effort to explore new partnerships among single older adults in Taiwan as well as their diverse attitudes toward establishing partnerships in later life.

## Method

### Design and participants

The study was conducted in two stages. In the first stage, using interview questions (Additional file 1), 10 in-depth interviews were conducted to explore the progress of developing partnerships among single older adults in order to construct concourses for social representations. In the second stage, we recruited 49 single older adults to complete the subjective array of Q statements [27]. This study was reviewed and approved by the Research Ethics Committee of National Taiwan University.

The first stage employed purposive sampling to recruit single older adults who have been involved in a new partnership for more than two years. Among the 10 recruited participants, six were female and four were male. Among the six women, two were divorced and the other four were widows. Among the four men, two were widowers, one was divorced, and one had been single. The length of their current partnerships ranged from 2 to 17 years; regarding their current marital status, one participant was married for the first time and two had re-married.

In the second stage, we recruited 49 new participants to participate in the Q sorting who met the following criteria: (a) aged 65 years or above, (b) single, (c) normal cognitive function, (d) engaged in a new partnership, and (e) willing to provide written informed consent. These 49 single older adults were recruited from three community organizations, five elderly apartment complexes, and six elderly care centers in Northern Taiwan. The mean age of the participants was 73.98 (*SD* = 7.71; range from 65 to 91 years) and comprised 22 males (45%) and 27 females (55%). Fifty-nine percent of the participants were widowed and 41% were divorced. Seventeen participants (35%) lived alone, 13 (27%) lived with a family member, and 19 (39%) lived in apartment complexes or care centers focused on the elderly.

### Development of Q statements

In first stage, the interview guidelines were sent to each participant prior to their face-to-face interview, which allowed the interviewee’s time to prepare. Each interview lasted between 1.5 and 2 h and was audio recorded and subsequently transcribed verbatim. All interviews were conducted in participants’ residences and in their native language.

**Fig. 1:**
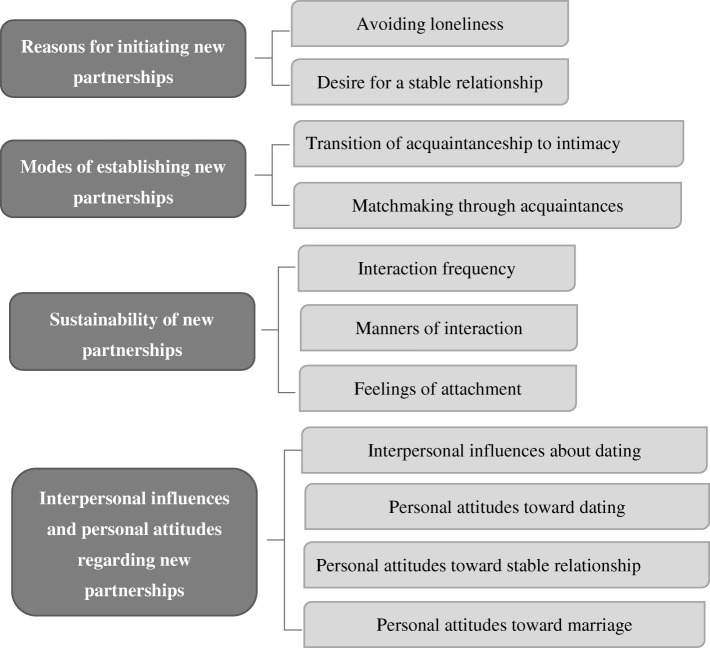
Experience of establishing a new partnership in later life

The major themes and categories are presented in Fig. 1. Q statements were constructed from the results of a content analysis, which revealed that participants’ perceptions could be divided into 11 categories; from these categories, four themes emerged. Based on a text analysis of the interview data, we extracted and developed 50 statements associated with partnership among older adults. After a review by three health professionals and a research team, 40 Q statements were adopted. The 10 participants from the first stage were recruited again for a pilot study to test the validity and reliability of the Q statements. Ambiguous and confusing statements were modified to ensure that the interviewees could comprehend all statements. The final sets of 40 Q statements were representative of the original verbiage used regarding partnerships among the 10 interviewed older adults.

### Q sorting

Q sorting of the statements was conducted in a disturbance-free location, where participants could select the statements independently and confidentially. This process lasted for approximately 50 min and was conducted individually by each participant. In this study, statement cards (each containing one Q statement) and a sorting grid were utilized as support tools for the Q sort. The participants were asked to sort and rank the Q statement cards onto the grid, which was divided into 9 columns, each representing a degree of importance. Participants would rank each statement according to their perception, ranging from “-4” (“least important”) to “4” (“most important”).

Following the Q sort, participants were interviewed to obtain further information about their sorting decisions, such as the disclosure of specific meanings they associated with any of the statements, or if there were statements that they thought to be of particular importance/ unimportance (with an emphasis on those ranked ±4). The audio-recorded interviews were then transcribed verbatim to allow participants’ comments to be used during the interpretation of the Q analysis.

### Q analysis and factor interpretation

The 49 participants who completed the Q sorting were inter-correlated and factor analyzed using the statistical software package PQ Method, version 2.35. A principal component analysis with a Varimax rotation was conducted to extract the relevant factors of all perspectives. We employed a combination of eigenvalues, which reflect variation accounted for by a corresponding factor, as well as a scree plot to determine the number of retained factors. We found that a five-factor solution was the best fit for the data, where each factor comprised at least four Q-sort loadings, which were high and significant (*p* < .05) on only one factor.

## Results

We performed a factor analysis on the rankings (Q sorts) of the 40 Q statements. Five factors were extracted from the data and the orthogonally rotated solution explained 53% of the overall variance. The scores for each statement across all five factors are displayed in Table 1, which highlights the corresponding theme, Q statements, and factor arrays. Table 2 shows the socio-demographic characteristics of the participants along the five factors. In the factor descriptions below, the parenthetical notations represent the statement rankings within the factor arrays; for example, “(01:4, 1.89)” means that Statement 1 is ranked in Position 4 (i.e., the most important position), and that the z-score is 1.89. Distinguishing statements at both extremes among participants loaded on the five factors are listed in Table 3.

**Table 1:** Q-Statements and Factor Arrays Across the Five Factors

Theme Q statement	Factor arrays				
	F1	F2	F3	F4	F5
Reasons for initiating new partnerships					
1. compensate for loneliness	2	0	4	3	1
2. partner should be single	4	2	**−2**	3	4
3. to operate within a long-term stable relationship	1	**3**	1	**-2**	1
Modes of establishing new partnerships					
4. partners should already know each other	**4**	**−1**	−3	2	− 3
5. partner should be introduced by my relatives and friends	0	−3	−4	**2**	0
6. partner is living nearby	**2**	0	−2	**−4**	0
Sustainability of new partnerships					
7. meet every day	1	−4	1	−3	−3
8. engage in common interests and hobbies	3	2	2	1	3
9. attend each other’s family activities	−1	0	−2	0	−2
10. the companionship of partners and family members are different	0	−1	**4**	0	2
11. make me feel young again	3	−1	2	2	0
12. enjoy the feeling of mutual companionship	3	4	2	1	2
13. enjoy the feeling of being hand-in-hand, embracing and kissing	0	**−3**	**3**	**−1**	2
14. satisfaction of sexual needs	−1	**−4**	0	−1	−2
15. assist to broaden the interpersonal network	0	0	0	0	2
16. enrich and share a life together	2	1	3	4	0
17. enjoy the romantic feeling that you never had when you were young or in a previous marriage	1	1	1	−1	− 1
18. enjoy a normal and tangible life	2	1	1	3	1
Interpersonal influences and personal attitudes regarding new partnerships					
19. consider the thoughts of adult children	−1	0	0	1	0
20. consider the thoughts of family members	−2	−2	−2	0	0
21. consider the thoughts of relatives and friends	−2	−3	0	**2**	−1
22. need to keep my own independence	0	1	2	0	**3**
23. is a proud and glorious matter	1	−2	−3	−2	1
24. has a certain risk	−2	0	−2	**2**	0
25. consider mutual age problems	0	0	−3	−2	**2**
26. consider mutual health conditions	1	3	2	4	4
27. consider mutual financial situations	−1	**3**	1	1	−1
28. consider mutual religious beliefs	−1	**1**	−1	−3	−2
29. live together after our relationship is stable	0	0	0	−2	− 2
30. give each other commitment after our relationship is stable	1	**2**	1	1	0
31. take care of each other after our relationship is stable	2	**4**	3	0	**−1**
32. share the living expenses after our relationship is stable	−1	2	0	1	**−4**
33. regard marriage as the ultimate purpose	−2	**1**	−4	−1	−4
34. consider mutual health situations, then decide whether to get married or not	0	2	−1	**−4**	3
35. consider mutual age problems, then decide whether to get married or not	**−3**	−1	−1	0	1
36. consider the problem of caring for children, then decide whether to get married or not	−3	−2	−1	− 1	−3
37. consider the problem of caring for parents, then decide whether to get married or not	**−4**	−1	− 1	− 2	**1**
38. consider mutual property succession problems, then decide whether to get married or not	−3	−2	0	0	−2
39. consider family problems, then decide whether to get married or not	−4	−2	0	−1	−1
40. consider the problem of handling housework, then decide whether to get married or not	−2	−1	−1	−3	− 1

^a^ Factor ratings were identified via a Q-sort factor analysis and indicate how statements were ranked (from + 4 [most important] to − 4 [the least important]) by participants who loaded significantly onto a given factor. ^b^ Boldface indicates the significance (*p* < .01) of the distinguishing statements

**Table 2:** Socio-demographic Characteristics with the Five Factors (*n* = 38)

Variables	F1 (*n* = 13)	F2 (*n* = 9)	F3 (*n* = 6)	F4 (*n* = 6)	F5 (*n* = 4)
	*n*, %	*n*, %	*n*, %	*n*, %	*n*, %
Age (M [SD])	77.08 (7.76)	73.44 (8.80)	71.67 (4.93)	72.83 (9.75)	71.00 (9.41)
Education (years) (M [SD])	11.54 (3.36)	12.00 (3.57)	12.33 (3.67)	10.00 (3.10)	12.50 (4.73)
Gender					
Female	5 (38%)	5 (56%)	4 (67%)	4 (67%)	2 (50%)
Male	8 (62%)	4 (44%)	2 (33%)	2 (33%)	2 (50%)
Marital status					
Widower/widow	7 (54%)	6 (67%)	3 (50%)	4 (67%)	0 (0%)
Divorcé/divorcée	6 (46%)	3 (33%)	3 (50%)	2 (33%)	4 (100%)
Living status					
Solitary	2 (15%)	1 (11%)	3 (50%)	1 (17%)	3 (75%)
With family member	3 (23%)	4 (44%)	0 (0%)	3 (50%)	1 (25%)
Facility	8 (63%)	4 (44%)	3 (50%)	2 (33%)	0 (0%)
Incomes					
Working salary	1 (8%)	1 (11%)	2 (33%)	0 (0%)	0 (0%)
Pension & savings	7 (54%)	7 (78%)	4 (67%)	5 (83%)	4 (100%)
Supported by children	5 (39%)	1 (11%)	0 (0%)	1 (17%)	0 (0%)

**Table 3:** Distinguishing Statements at Both Extremes across the Five Factors

	Important level	F 1	F 2	F 3	F 4	F 5
Reasons	Most		3. operate within a long-term stable relationship (3)			
	Least					
Modes	Most	4. partners should already know each other(4)				
	Least				6. partner is living nearby (−4)	
Sustainability	Most			10. the companionship of partners and family members are different (4) 13. enjoy the feeling of being hand-in-hand, embracing, and kissing (3)		
	Least		13. enjoy the feeling of being hand-in-hand, embracing, and kissing (−3) 14. satisfaction of sexual needs (−4)			
Personal attitude & Interpersonal influences	Most		31. take care of each other after our relationship is stable (4) 27. consider mutual financial situations (3)			22. need to keep my own independence (3)
	Least	37. consider the problem of caring for parents, then decide whether to get married or not (−4) 35. consider the mutual age problem, then decide whether to get married or not (−3)			34. consider mutual health problems, then decide whether to get married or not (− 4)	32. share living expenses after our relationship is stable (− 4)

### Factor 1: Being single, a companion, and already acquaintances

This group of perspectives accounted for 15% of the total variance, with an eigenvalue of 13.99. Participants loaded in Factor 1 emphasized the concerns of being single and already an acquaintance to each other before establishing a partnership. These participants emphasized companionship and sharing common interests; examples of the statements in this factor include “partner should be single” (02: 4, 1.72), “partners should already know each other” (04: 4, 1.42), “enjoy the feeling of mutual companionship” (12: 3, 1.42), “engage in common interests and hobbies” (8: 3, 1.37), and “make me feel young again” (11:3, 1.31). Participants of this factor also claimed that they did not necessarily consider the perspectives of parents, children, or other family members, as reflected in the following statements that they rated as least important: “consider the problem of caring for parents, then decide whether to get married or not” (37: − 4, − 1.70); “consider family problems, then decide whether to get married or not” (39: − 4, − 1.36); “consider mutual age problems, then decide whether to get married or not” (35: − 3, − 1.35); “consider the problem of caring for children, then decide whether to get married or not” (36: − 3, − 1.24). Statements 4, 35, and 37 were major distinguishing statements of participants loaded on this factor compared to participants in other factors; for example, participant No. 27 said the following:

*Although we knew each other, we did not have further interaction until we met in a community activity after our spouses had passed away. We are both older now and we cherish this opportunity to hang out together. It is nice to have someone in late life without having to consider marriage and family burdens. (Female, 70 years old)*

Overall, the participants in this factor had the highest mean age (*M =* 77.08, *SD* = 7.76) among the five factors; furthermore, the majority (63%) lived in an elderly facility. It is possible that these participants no longer view marriage as necessary; rather, that they would like to have a partner—who was already an acquaintance—to share common interests and enjoy activities together, without pressure to getting married.

### Factor 2: High spiritual compatibility and caring personality

This factor accounted for 9% of the total variance and had an eigenvalue of 3.81. Participants loaded on this factor emphasized looking after one another and pursuing a long-term partner. They considered the following statements as highly important: “take care of each other after our relationship is stable” (31: 4, 2.04), “enjoy the feeling of mutual companionship” (12: 4, 1.51), “operate within a long-term stable relationship” (3: 3, 1.46), and “consider mutual religious beliefs” (28: 1, 0.68). Participants in this factor considered “satisfaction of sexual needs” as the least important component of a relationship (14: − 4, − 2.38), suggesting that they did not consider sexual gratification as a necessary component of partnership. They also did not think it was important to see their partner every day or have intimate physical contact, as indicated by their rankings for “meet every day” (7: − 4, − 1.74) and “enjoy the feeling of being hand-in-hand, embracing and kissing” (13: − 3, − 1.52). Aside from the main distinguishing statements above (3, 13, 14, 27, and 31), they regarded the statement, “regard marriage as the ultimate purpose” (33: 1, 0.50) as rather important compared to participants in other factors; for example, participant No. 28 said the following:

*I think having a partner relationship means to look after each other. Over the past three years, my memory has been deteriorating; my hands have begun to shake. Fortunately, I have my partner to rely on and take care of me even if we don’t live together now. (Male, 78 years old)*

Compared with participants in other factors, those loaded on Factor 2 had the highest percentages of being widowed (67%). Given that these participants did not feel the need to meet every day or engage in regular intimate physical contact, the key characteristic of the participants in this group was to establish a long-term partner relationship such as getting married and taking care of each other.

### Factor 3: Emphasis on physical intimacy and companionship

This factor accounted for 12% of the total variance and had an eigenvalue of 3.26. Participants loaded on this factor believed that partnership is an effective strategy to avoid feelings of loneliness in their lives and this allows them to enjoy their partner’s companionship. The statements that they considered important included “to compensate for loneliness” (1: 4, 1.89) and “the companionship of partners and family members are different” (10: 4, 1.88). Furthermore, participants enjoyed physical contact in their partnerships, such as “enjoy the feeling of being hand-in-hand, embracing, and kissing” (13: 3, 1.51), but they did not consider it necessary to be familiar with the partner before establishing the relationship, to be introduced by friends or relatives, or feel the need to get married. Statements that they considered unimportant included “introduced by my relatives and friends” (5: − 4, − 1.88), “partners should already know each other” (4: − 3, − 1.55), and “regard marriage as the ultimate purpose” (33: − 4, − 1.79). Overall, their main distinguishing statement was “partner should be single” (2: − 2, − 0.70), followed by statements 10 and 13; for example, participant No. 18 said the following:

*After getting divorced and retired, the emptiness in the day and loneliness in the night had been corroding my soul ... Over the 18 years since being with her (my current partner), we have been there for each other, which has removed the loneliness from our lives and made us feel young again ... I do not want to get married, because usually, after getting married, women tend to have lots of requirements and expectations of their husbands. I do not want her to worry about such things, so not getting married is the best status for both of us. (Male, 78 years old)*

The participants loaded on this factor had a younger mean age than in other factors (*M* = 71.67, *SD* = 4.93). These participants emphasized physical intimacy in a relationship and did not care whether the potential partner was single or not; furthermore, they believed that late-life partnerships should be enjoyed for purposes of companionship, without considering marriage.

### Factor 4: Easily influenced by others’ comments and highly concerned

This factor accounted for 7% of the total variance and had an eigenvalue of 2.83. Participants loaded on this factor were afraid of loneliness and being bored, and sought new partnerships to enrich and share a mutual life; however, they also felt influenced by the perspectives of relatives and friends and believed that there were risks involved with establishing a new partnership. The most important statements for these participants were “consider mutual health conditions” (26: 4, 2.08), “enrich and share mutual life” (16: 4, 1.52), “compensate for loneliness” (1: 3, 1.34), and “enjoy a normal and tangible life” (18: 3, 1.41). They disagreed with the statements “partner is lives nearby” (6: − 4, − 2.52) and “consider mutual health conditions, then decide whether to get married or not” (34: − 4, − 1.97). The additional major distinguishing statements of participants loaded on this factor were “has a certain risk” (24: 2, 0.97); “consider the thoughts of relatives and friends” (21: 2, 0.83); and “partner should be introduced by my relatives and friends” (5: 2, 0.82); for example, participant No. 19 said the following:

*Since my husband passed away, even though my friends had been encouraging me to find a boyfriend to date that can enrich my life, as I was concerned about the perspectives of neighbors. They might criticize me to find a new partner so soon after my husband passed away. I have no objection toward pursuing a new partner, but I am afraid of being criticized and cheated for wealth or purity. (Female, 67 years old)*

Compared with their counterparts in other factors, these participants had a lower education level (*M* = 10.00, *SD* = 3.10). These participants were characterized by worrying about taking a risk when engaging in new partnerships although they were also afraid of loneliness; furthermore, they preferred their partner to be introduced by relatives and friends, and were concerned about comments that might be made by acquaintances.

### Factor 5: Physical and financial Independence

This factor accounted for 10% of the total variance, with an eigenvalue of 2.52. The participants loaded on this factor considered that “partner should be single” (2: 4, 1.97) as the most important factor for establishing a partnership. They further regarded “consider mutual health conditions” (26: 4, 1.96) and “need to keep my own independence” (22: 3, 1.94) as relatively important. In contrast, the statements that they considered least important were “share living expenses after our relationship is stable” (32: − 4, − 1.79), “regard marriage as the ultimate purpose” (33: − 4, − 1.75), and “partners should already know each other” (4: − 3, − 1.66). Another distinguishing statement regarded as unimportant was “take care of each other after our relationship is stable” (31: − 1, − 0.64). The main distinguishing statements for participants in this factor were statements 22, 32, and 31; thus, participants loaded on this factor got involved in a relationship from the perspective of pursuing a partner, and felt that they and their partner were independent in terms of health and finances. They did not consider marriage a viable option; for example, participant No. 17 said the following:

*I think having a new partnership is the only way to slow down the aging process. Therefore, before getting involved in partnership, it is very important that one can love oneself first, enjoy life, and maintain independence and autonomy, both physically and economically. I have had the experience of marriage, and know what marriage looks like. Establishing a new partnership is enough for me, there’s no need to consider getting remarried in the future. (Female, 68 years)*

Unlike their counterparts in other factors, all participants loaded on this factor were divorced, with the majority living alone (75%) and subsisting primarily on their own pension and/or savings.

## Discussion

The present study adopted the Q methodology to investigate single older adults’ perspectives on new partnerships. Five factors were identified, which were primarily differentiated in terms of perspectives regarding the reasons for and modes of establishing new partnerships, the sustainability of such partnerships, and interpersonal influences and personal attitudes concerning these partnerships.

Participants loaded on Factor 1 were the oldest among all participants. Löckenhoff and Carstensen [28] indicate that when older adults become aware of their limited time left in late life, they prefer emotionally gratifying social partners over novel social contacts. In line with this finding, older adults loaded on this factor were more interested in finding a single companion with common interests and hobbies from among their acquaintances and neighbors rather than new individuals with superfluous interests. Overall, the companion relationships discussed by those in Factor 1 comprised multiple dimensions; for example, there are diverse companion relationships to label as “partner” such as “special friend,” “a boy/girlfriend,” and “a fine mate.” The characteristics of companion relationships are long-term and involve a clear commitment to joint activities such as common interests and hobbies [13]. A unique finding of this study was that participants would prefer to select their partners from their acquaintances and neighbors, which has rarely been reported in prior literature. This attitude might be attributed to the fact that they all belong to a certain center or facility that provides opportunities to interact with acquaintances and neighbors.

A second look at participants loaded on Factor 1 is that they felt young again as a result of operating with a new partnership, without considering marriage or factors related to it. This is expected to contribute to improved self-image and youthful behaviors, such as walking around arm-in-arm [15]. Older adults who feel young again will be able to experience romantic feelings and construct the self-image of a much younger adult, which should contribute to perceptions of their old age in a manner different form normative expectations.

Most of the participants loaded on Factor 2 were widowers and widows who desired to have a long-term partner with whom they could have a companionship where they could take care of each other, rather than someone with whom they can meet every day and engage in sex or intimate physical contact. Most of the participants were careful to explain that sexual relations were not a major part of their partnerships. They explained that, in later life, sex does not matter as much as when they were at a younger age [29], but that companionship and commitment are important [7, 12]. However, as indicated in prior studies [30], older women demonstrated a diverse attitude regarding their desire for sex as well as the types of sexual activities in which they would like to engage. The need for sex and intimate physical contact of divorced or separated women may differ from those who are windowed or never partnered. On the contrary, the older adults of Factor 3 tend to perceive that the building of new partnerships can enjoy the feeling of physical intimacy. DeLamater and Moorman [31] also explains that although age will affect the performance of sexual behavior, the nature of the older adults toward the sexual presentation indeed reflects the interaction of body, mental and social background.

It has been shown in prior literature [32] that financial strain influences the relationship of older couples; as such, it is not surprising to find that participants in this present study, who were loaded on Factor 2, considered mutual financial situations as a key element of new partnerships. The variables of education, financial security, and health have been shown to be directly related to remarriage of older adults, with financial security as a critical component in the management of long-term partnerships. For those participants in the present study who disclosed a desire for a long-term, stable relationship, it is practical for them to consider their mutual financial situations [33]. Compared to their counterparts loaded on other factors, a distinguishing characteristic of participants in Factor 2 is that they desired to get married once their relationship was more stable.

One important characteristic for the older adults loaded on Factor 3 was that they did not depend on their children’s financial support; in addition, they indicated that the companionship of partner and family members are different and they did not care whether their partner was single or not. They enjoy the feeling of being hand-in –hand, embracing, and kissing. To them, partnerships refer to relieving feelings of loneliness and enjoying physical intimacy [34]. Bender, Burgess and Barmon [35] indicate that although the older adults still have desire for sex and partnership, they are possibly limited by environment (for example, Assisted Living) and result in the limitation of availability of and access to desirable partners and their personal privacy, and lead to the prohibition of facility and gossips from others. In some cases, they enact strategies (i.e., excuses, justifications, and active dismissal of desire) to remove desire from the equation, especially when facing barriers. Therefore, facility’s policy and environment should consider the feeling need of the older adults to make adjustment, and staff training also needs to reinforce the acknowledge and understanding toward the close relationship of the older adults. For the general public, they should face the desire of the elders toward sex and partnership with more open attitude.

Participants loaded on Factor 4 were more concerned than their counterparts about the viewpoints of others who were close to them. These participants comprised the lowest level of educational attainment and the highest percentages of widowers/widows, and that most were living with a family member and subsisting on income from a pension and/or savings. Intertwining a desire to establish a partnership and a fear of taking risks, the participants on this factor believed that it is best to find a partner through their friends and family members’ recommendation—a unique finding that has rarely been reported in prior literature. These older adults did not expect to see their partner daily (7: − 3, − 1.52) or that they even had to live nearby (6: − 4. -2.52). Unlike their counterparts in other factors, they did not plan to operate within a long-term relationship (3: − 2, − 1.17). In line with Määttä [14], the results of the present study indicated problems of prejudice from close others in their environment, which can cause older adults to hide their relationships to avoid gossip.

Participants loaded on Factor 5 were all divorced, and had the highest percentage of participants living alone while having income from pension and savings. These participants preferred that their partner was single and that they could be independent without having to take care of each other; as such, mutual health conditions and financial security were considered critical conditions of partnership. These findings coincide with a qualitative study conducted by Watson and Stelle [12], who showed that some elderly women did not consider marriage the ultimate goal of establishing a new partnership; they simply would like to enjoy the feelings that come with being in love. Fileborn et al. [30] further pointed out that women expected romance and sexual intimacy within a relationship, but wanted to protect their independence and were reluctant to enter into a new relationship later in life, due in part to fears of falling into the role of caregiver and housekeeper [12, 18]. Among men, particularly divorcés, there was a desire to experience freedom from the bonds of marriage, and possibly from any future divorce-related costs (e.g., alimony and property division). Therefore, they would like to continue being economically independent even after stabilizing the relationship.

It is critical for older adults to be self-aware about which factor they were loaded on as well as about their own attitudes toward partnership before they get involved with someone new. Results from this study show that older adults tend to pursue partners with similar viewpoints in the hope of avoiding conflicts generated by differences in perspectives and values, thus ensuring a turbulence-free long-term relationship.

This study explored older adults’ diverse perspectives regarding the reasons for initiating new partnerships, modes of establishing these partnerships, sustainability of partnerships, and interpersonal influences and personal attitudes regarding partnerships. Health professionals serve a vital role in providing care to older patients and residents in long-term care facilities; as such, they could provide valuable suggestions to satisfy older adults’ need for companionship and help to improve their mental health and quality of life through new partnerships [36]. If health professionals possess sufficient knowledge and understanding regarding the issue of partnership among older adults, they can display attitudes of solicitude, empathy, and openness to discussing diverse behavior patterns and adjustment issues during the establishment of new partnerships.

Our study provides a starting point for future research in the area of older adult partnership in Taiwanese society and, particularly, five factors of perspectives that are common among those individuals seeking late-life partnerships. Critical reflection on older adults’ own perspectives and interpretations of this responsive approach is a key element for appropriating support for the formation of new partnerships. According to which perspective the older adult desires a new partnership (e.g., which of the five factors), elderly care practitioners, family members, and caregivers can be better aware of what kind of approach may be required to encourage them to establish a new partnership.

This study describes the attitudes of single older adults toward partnership in various categories. Nonrandom sampling of participants may hinder applicability of the findings to other populations. However, the Q methodology was useful for the exploration of diverse patterns of thought rather than prevalence of views. Moreover, no comparisons or correlations were made between the factor a participant sorted to and one of his/her characteristics (e.g., age, being widowed, living close to a partner, etc.). In the future, when assessing the needs of older adults, our results would be helpful to provide advice, which will benefit the quality of health services. Moreover, our findings provide the basis for the future development of scales and APP, and help older adults looking for a partner with the adapted method, which is more efficient.

## Conclusion

This study adopted the Q methodology to investigate single older adults’ perspectives on new partnerships. Five factors were identified, which were primarily differentiated in terms of their perspectives on the reasons for and modes of establishing partnerships, the sustainability of the partnerships, and the interpersonal influences and personal attitudes concerning the partnerships. The findings of this study can provide researchers and practitioners with critical information in dealing with older adults’ attitudes toward new partnerships. It is only through an understanding of late-life partnerships that caregivers and practitioners can engage in successful consultations with older adults who desire to have a new partnership.

## Additional file

Additional file 1: Interview questions. This document contains the list of questions. (DOCX 12 kb)
