# Supplementary material for: New partnerships among single older adults: a Q methodology study
Source: BMC Geriatr. 2019 Mar 6;19:74. doi: 10.1186/s12877-019-1091-5 (PMC6404340; doi:10.1186/s12877-019-1091-5)
Supplement: Supplementary file 1 — Interview questions. This document contains the list of questions. (DOCX 12 kb) [file 12877_2019_1091_MOESM1_ESM.docx]

The interview questions are described as follows:

1.How did you and your partner meet?

2.Why did you choose him (her) to be your partner?

3.How do you interact?

4.How has this partnership influenced your everyday life?

5.Have you encountered obstacles during the relationship?

6.What is your prospect of this relationship?
